# Siamese network with a depthwise over-parameterized convolutional layer for visual tracking

**DOI:** 10.1371/journal.pone.0273690

**Published:** 2022-08-31

**Authors:** Yuanyun Wang, Wenshuang Zhang, Limin Zhang, Jun Wang

**Affiliations:** 1 School of Information Engineering, Nanchang Institute of Technology, Nanchang, Jiangxi, China; 2 Jiangxi Province Key Laboratory of Water Information Cooperative Sensing and Intelligent Processing, Nanchang Institute of Technology, Nanchang, Jiangxi, China; Kingston University, UNITED KINGDOM

## Abstract

Visual tracking is a fundamental research task in vision computer. It has broad application prospects, such as military defense and civil security. Visual tracking encounters many challenges in practical application, such as occlusion, fast motion and background clutter. Siamese based trackers achieve superior tracking performance in balanced accuracy and tracking speed. The deep feature extraction with Convolutional Neural Network (CNN) is an essential component in Siamese tracking framework. Although existing trackers take full advantage of deep feature information, the spatial structure and semantic information are not adequately exploited, which are helpful for enhancing target representations. The lack of these spatial and semantic information may lead to tracking drift. In this paper, we design a CNN feature extraction subnetwork based on a Depthwise Over-parameterized Convolutional layer (DO-Conv). A joint convolution method is introduced, namely the conventional and depthwise convolution. The depthwise convolution kernel explores independent channel information, which effectively extracts shallow spatial information and deep semantic information, and discards background information. Based on DO-Conv, we propose a novel tracking algorithm in Siamese framework (named DOSiam). Extensive experiments conducted on five benchmarks including OTB2015, VOT2016, VOT2018, GOT-10k and VOT2019-RGBT(TIR) show that the proposed DOSiam achieves leading tracking performance with real-time tracking speed at 60 FPS against state-of-the-art trackers.

## 1 Introduction

Visual tracking is an important research topic in computer vision. In visual tracking, some key techniques are applied, such as machine learning [1, 2], image segmentation [3] and object detaction [4]. It has a variety of tasks such as video surveillance, automatic driving and human-computer interaction. Although much progress has been made recently, it is still a challenging task due to the influence of various factors such as scale variation, in-plane rotation, motion blur, out-of-plane rotation and fast motion.

Visual tracking aims to predict the target locations in subsequent frames, given the initial target state in the first frame. Existing trackers can be roughly divided into two kinds. The algorithms in the first kind use correlation filter technique to address the correlation between the target template and search regions [5, 6]. The position of the maximum response value is the position of the target in the current frame. KCF [7] further extends circulant matrix and uses muti-channel feature maps for high speed tracking. To solve scale variations, DSST [8] learns discriminative correlation filters based on a scale pyramid representation to handle large scale variations. Correlation filter based algorithms have low computational cost and obtain real-time tracking performance.

In another kind, algorithms aim to extract deep features by CNN for target representations [9, 10]. Recently, trackers based on CNN [11, 12] have great progress due to the strong feature representational ability. Tracking algorithm [13] fully uses features from different CNN layers and facilitates end-to-end training of the trackers. The tracking algorithm [14] uses CNN for feature extraction to distinguish target from distractors. In order to fully exploit the feature representation power of CNN, MDNet [15] learns domain-independent representations by trained CNN and captures domain-specific information to track targets. For getting accurate target state estimation, ATOM [12] uses the combination of target estimation and classification to receive accurate bounding boxes. The key to design high-performance trackers is to find expressive features and corresponding classifiers. In order to improve the tracking speed and tracking accuracy, Valmadre *et* *al*. [16] combine the CNN features and correlation filter.

Recently, Siamese network is widely used in visual tracking [17, 18]. Siamese network based trackers formulate visual tracking as a similarity matching problem between the target template and search regions. SiamFC [9] achieves leading tracking performance as well as in balancing the accuracy and speed. DSiam [19] uses multi-level deep feature fusion to integrate network output, and obtains the target appearance change information. Based on SiamFC, such as DaSiamRPN [20] and SiamBAN [21], some trackers improve tracking accuracy by using different Siamese network frameworks.

Due to the complex network characteristics of CNN, several Siamese networks have been trained offline [22, 23] to improve the feature extraction power. Currently, AlexNet [24], ResNet [25] and VGG-M [26] are used in visual tracking as backbone networks. Feature extraction is an important component in visual tracking. Existing trackers [27, 28] extract features by conventional convolution layers. Although existing trackers achieve excellent tracking performance, the spatial structure information are not been fully exploited in conventional convolution, which are important and beneficial for locating targets.

In order to obtain a robust feature representation for visual tracking, in this paper, inspired by the depthwise over-parameterized convolution layer [29], we design a novel feature extraction subnetwork. The subnetwork consists of depthwise over-parameterized and conventional convolutional layers. Based on the proposed feature subnetwork, we propose a novel tracking algorithm in Siamese framework. The proposed DOSiam effectively utilizes the feature extraction ability to discard much adverse background information. Extensive experiments conducted on five benchmarks show that the proposed DOSiam tracker has outstanding tracking performances. Compared to conventional convolution methods, DOSiam shows that depthwise over-parameterized convolutional layer improves the representation power of targets.

The main contributions are summarized as follows:

- We propose a feature extraction subnetwork with the depthwise over-parameterized convolutional layer for extracting the more detailed target information by exploiting spatial structure and semantic information in Siamese-based tracking framework.
- We design a novel tracking algorithm by integrating feature extraction subnetwork, cross-correlation operation and tracking head. DO-Conv based feature extraction subnetwork adaptively focuses on shallow spatial and deep semantic information, and better locates targets.
- Extensive experimental results on challenging benchmarks including OTB2015, VOT2016, VOT2018, GOT-10k and VOT2019-RGBT(TIR) show that the proposed tracker performs better than the state-of-the-art algorithms and improves tracking performance.

This paper is organized as follows. Section 2 introduces related works. Section 3 describes the proposed tracking algorithm. Section 4 presents the experimental results. Finally, we draw a conclusion in Section 5.

## 2 Related works

We mainly review some representative tracking algorithms and techniques related to the proposed tracker, including Siamese network based tracking algorithms, feature extraction and fusion methods in this section.

### 2.1 Tracking based on Siamese network

In recent years, Siamese based trackers have drawn great attention for their balanced accuracy and speed. In Siamese based trackers [30, 31], a similarity function is learnt for measuring the similarity between the target template and search regions. Many significant works consider SiamFC as a baseline to improve the tracking performance. In order to use the semantic information, He *et* *al*. [32] design a twofold branch tracking framework, including a semantic branch and an appearance branch. In SiamDW [33], Zhang *et* *al*. design a lightweight backbone to improve the capability of deep neural networks from three aspects, including receptive field size, network padding and stride.

Inspired by the region proposal network for object detection [34], SiamRPN gets benefit from using the output of Siamese network to perform the region proposal extraction. By jointly learning a classification branch and a regression branch for region proposal, SiamRPN extracts feature maps and locates objects with high performance. Zhu *et* *al*. [20] address imbalanced distribution of training data by effective sampling strategy, and design a distractor-aware module to perform an incremental learning. In order to address accuracy gap with lack of strict translation invariance, Li *et* *al*. [35] design a simple and effective spatial aware sampling strategy. SiamRPN++ [36] randomly shifts the training object location in search regions during training to eliminate the center bias. It has a significant performance gain.

Guo *et* *al*. [37] propose an effective anchor free framework with Siamese classification and regression. In the field of unmanned aerial vehicle (UAV) [38], the trackers track small targets with numerous background interference. In order to solve the above problems, Huang *et* *al*. [39] propose a robust Siamese network tracker based on spatio-temporal attention. It implements local tracking and re-detection alternatively. The Siamese network framework is also widely used in infrared target tracking. In order to alleviate target drifting, Xu *et* *al*. [40] propose an adaptive Siamese network based on hierarchical convolution fusion network, which combines shallow spatial and deep semantic information. This tracker achieves high tracking performance.

To obtain better the effectiveness of feature fusion, Chen *et* *al*. [41] present a novel attention-based feature fusion network that effectively embeds the target template and search regions features by using attention. TransT [41] takes the advantages of self-attention and cross-attention. Wang *et* *al*. [42] propose a valid Siamese network architecture that has two parallel branches, namely, transformer encoder and transformer decoder to robust target tracking.

### 2.2 Feature extraction and fusion

Due to the strong expression ability, CNN has recently drawn a lot of attention in visual tracking [13, 43]. Convolutional layers are the core components of CNNs. We make full use of this to change the convolutional mode in conventional convolution networks. The backbone of Siamese network combines the targe template and the search regions into a embedding space. CNN makes great contributions to solving visual tracking problem. CNN is important for visual tracking to improve the feature extraction ability. The innovation of convolutional ways can better improve the tracking precision. Convolutional neural network mainly includes conventional convolution layers, which are the key for feature extraction. The target representation capability of convolutional neural network needs to be further improved. In order to better weight the deep feature information and improve the deep semantic information of the target, we design a feature extraction subnetwork to extract more abundant target feature information. The feature extraction subnetwork fuses conventional convolution layers and a depthwise over-parameterized convolution layer.

Feature fusion is an important component in Siamese based trackers, which evaluates the similarity between the targe template and search regions [44, 45]. Most of Siamese architectures assemble two branches by using the coarse naive correlation [20, 46] or depth-wise correlation [36, 44]. The cross-correlation module is used to embed a pair of convolutional features computed from Siamese networks. SiamFC [9] utilizes a cross-correlation model to obtain a response map for locating targets. The position with the maximum score corresponds is the positions of tracked targets. Li *et* *al*. [46] add a huge convolutional layer to scale the channels.

Wu *et* *al*. [36] use a depthwise cross correlation layer (DW-XCorr) to obtain efficient information connection. In order to maintain much spatial information, Yan *et* *al*. [47] consider pixel-wise correlation suitable for Alpha-Refine. In a refinement module, pixel-wise correlation considers every part of the target features as a kernel to avoid feature blur. To avoid neglecting the target structure information, Guo *et* *al*. [48] propose a simple target-aware Siamese graph attention network to match global feature for visual tracking. Different from the tracking algorithms above-mentioned, we propose a novel tracking algorithm based on a depthwise over-parameterized convolutional layer for visual tracking. It uses the depthwise over-parameterized convolutional layer(DO-Conv) to perform convolution operation for feature extraction.

## 3 Method

In this section, we will describe the proposed DOSiam tracker. DOSiam includes three essential components, namely feature extraction subnetwork, feature fusion and tracking head. Firstly, we design the feature extraction subnetwork that includes conventional convolution and depthwise over-parameterized convolution layers. Then, we will analyze the depthwise over-parameterized convolution layers for tracking. Finally, we will outline the DOSiam tracker.

### 3.1 Feature extraction subnetwork

Deep neural networks [14, 49] have been proven more effective in visual tracking. Siamese based trackers extract the target template and search region features by conventional convolution layers. In the conventional convolution operation, the channel independence and spatial information are not fully exploited. In visual tracking, the semantic and spatial information are helpful to represent a target.

In order to address the above problem, inspired by DO-Conv, we design a depthwise over-parameterized feature extraction subnetwork for visual tracking. DO-Conv combines conventional convolution and depthwise convolution operations. In the conventional convolution operation of DO-Conv, the spatial information of different channels is fused. DO-Conv is a combined of a depthwise convolution and a conventional convolution and refines information of targets. Depthwise convolution makes effective use of the channel feature information. The feature extraction subnetwork with DO-Conv makes fully use of the information of different channels, and improves deep feature learning capability and efficiency. The multilayer linear operations can be folded into a single layer operation followed the training phase. The computation is not increased. The extracted features contain abundant spatial and semantic information of targets. DO-Conv has wide usability and can be directly applied to the network training of existing trackers without requiring additional training or modifying other parts. In DOSiam, we use the modified AlexNet with DO-Conv as backbone network for feature extraction.

### 3.2 DO-Conv for tracking

**Fig 1:**
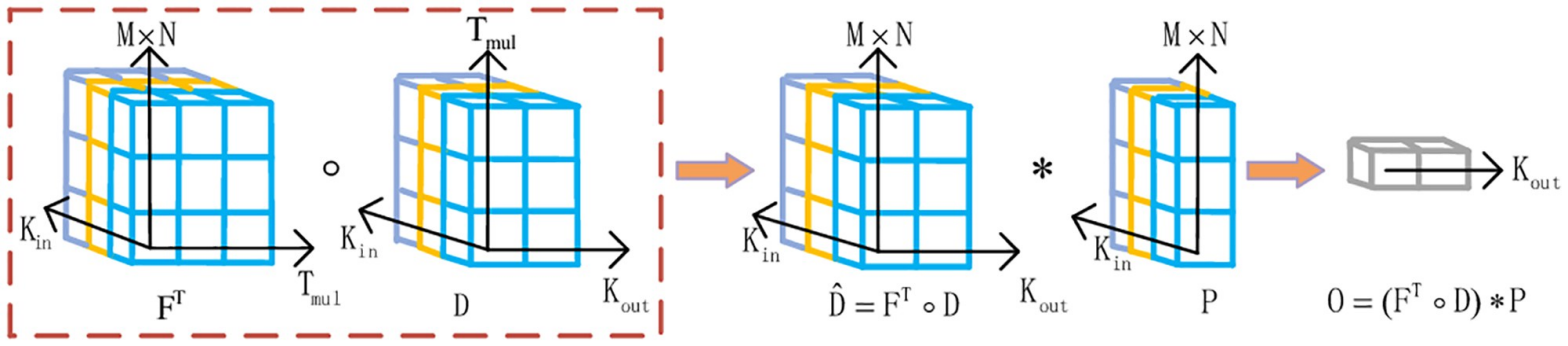
Illustration of DO-Conv. The deep convolution and conventional convolution kernel are included in DO-Conv. ∘ means the depthwise convolution operator and * means convolution operator.

DO-Conv consists of depthwise and conventional convolution kernels, as shown in Fig 1. Depthwise convolution kernel is denoted as $F\in R^{(M\times N)\times T_{\text{mul}}\times K_{\text{in}}}$. Conventional convolution kernel is denoted as $D\in R^{K_{\text{out}}\times T_{\text{mul}}\times K_{\text{in}}}$. $T_{\text{mul}}$ is referred as deep multiplier, where $T_{\text{mul}}\geq M\times N$. The receptive field of DO-Conv is M × N. $K_{\text{in}}$ is the number of channels in the input feature map. $K_{\text{out}}$ is the number of channels in the output feature map. An input patch is denoted as $P\in R^{(M\times N)\times K_{\text{in}}}$. The depthwise convolution operation is denoted as follow

$$\begin{array}{c} \hat{D}=F^{T}\circ D, \end{array}$$ (1)

where $\hat{D}$ is the results of depthwise convolutional operation. The output feature dimension is the same as the conventional convolution layer. The operation for DO-Conv can be expressed as follow

$$\begin{array}{c} O=(F^{T}\circ D)*P, \end{array}$$ (2)

where O is output of DO-Conv. (.)^T^ means the transposition matrix.

F^T^ performs depthwise convolution with the conventional convolution kernel D. Then, the result performs conventional convolution with P. After the training phase, the multilayer linear operations of F^T^ and D can be folded into a single operation. In the convolutional network, F and D can be optimized with the Stochastic Gradient Descent. DO-Conv not only accelerates the training process of the fully convolutional networks, but also achieves substantial progress in visual tracking. Although DO-Conv is used in feature extraction subnetwork, the computational complexity does not increase while the calculation of DO-Conv is similar to depthwise separable convolution in inference.

In addition, there are some differences between DO-Conv and depthwise separable convolution layer. Depthwise separable convolution is a combination of depthwise convolution and pointwise convolution. DO-Conv is a combination of depthwise and conventional convolutions. DO-Conv aims to speed up training with more parameters and achieves the best performance, simultaneously. DO-Conv has strong flexibility in feature extraction subnetwork, so it is easy to replace the conventional convolution layer in CNN.

### 3.3 DOSiam tracking framework

**Fig 2:**
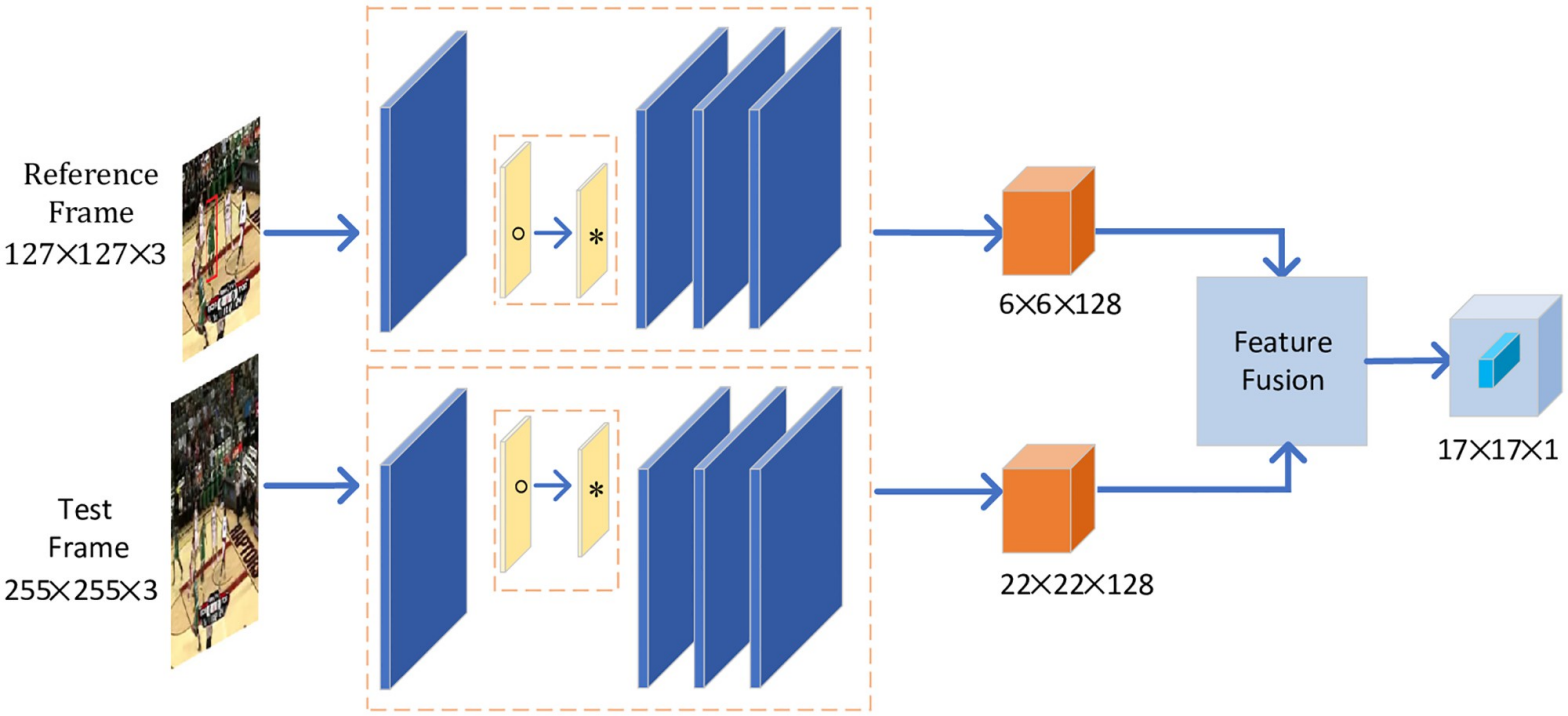
Illustration of DOSiam. It consists of the feature extraction subnetwork and cross-correlation operation. The feature extraction subnetwork contains conventional convolution layers and DO-Conv.

Based on DO-Conv, we design a novel feature extraction subnetwork in Siamese tracking framework, as shown in Fig 2. There are two branches in Siamese network. One is the template branch, where the only input is the target template. The other is the search branch with search regions as input image patches. We use z and X to represent the target template and search regions, respectively. The size of the target template is 127 × 127, while the size of the search region is 255 × 255. In Siamese network framework, the parameters are shared. The two branches of DOSiam are jointly trained together for real-time visual tracking. After feature extraction of each branch, feature vectors are obtained.

We apply the designed feature extraction subnetwork with DO-Conv to Siamese network framework. The subnetwork improves the convolutional capability and the feature extraction ability of Siamese network framework. Fully convolutional Siamese network with DO-Conv enhances the representation of targets appearance variation. The feature subnetwork further extracts the shallow spatial information, and reduces the loss of semantic information of the target template and search regions.

Feature fusion is a core component in Siamese-based tracking framework. It is an essential operation to calculate correlation of feature vectors in two branches. The proposed tracking algorithm utilizes a cross-correlation layer to receive response map for target location. Through feature fusion, the tracker obtains response maps as follow:

$$\begin{array}{c} h(z,X)=\text{corr}(f_{a}(z),f_{a}(X)), \end{array}$$ (3)

where $f_{a}(z)$ and $f_{a}(X)$ represent the feature maps of the template branch and search regions, respectively. The function corr(⋅) calculates the similarity of the target template and search regions.

The feature maps of the two branches have the same number of channels, and they execute cross correlation operation. By feature fusion, DOSiam gets a response map between the target template and search regions.

## 4 Experiments

### 4.1 Implementation details

The DOSiam is tested on the NVIDIA GeForce GT730 windows10 environment, with pytorch1.4.0. The backbone network of DOSiam is initialized with weights which is trained on GOT-10k [50]. GOT-10k is a large high-diversity benchmark. It contains 560 kinds of moving targets and provides 10,000 video sequences, which includes 1500,000 manually annotated bounding boxes. The video sequences in the training dataset and testing dataset have no overlap. The tracker DOSiam can trained and the training parameters are constantly adjusted. In feature extraction subnetwork, DO-Conv is a composition of conventional convolution and depthwise convolution operation. Conventional convolution kernel and depthwise convolution kernel are optimized by the gradient descent optimizer. In training, the number of parameters is increased in the linear transformation, but the feature extraction subnetwork with DO-Conv accelerates the training. In inference, the number of parameters DO-Conv is same of conventional convolution layer, and by experimental evaluation, DOSiam boosts the performance of the converged tracking framework. DOSiam achieves the state-of-the-art performance and runs at an average of 60 FPS, and higher than SiamFC which we run.

The input dimension of the DOSiam is the same as the baseline SiamFC. The feature maps of two branches have same number for channels. Through the feature extraction of DOSiam, the feature dimension of the target template is still 6 × 6 × 128 and the feature dimension of search regions are still 22 × 22 × 128. The tracking framework outputs response maps with 17 × 17 × 1, which are up-sampled 272 × 272 × 1. The training batch sizes are set to 8 and epoch numbers are set to 60. The learning rates of trained models are decreased from 0.05 to 0.00005.

### 4.2 Ablation study

#### DO-Conv indifferent AlexNet layers

We evaluate the performances of the depthwise over-parameterized convolutional layer in DOSiam. We verify the effectiveness of feature extraction subnetwork with DO-Conv. The designed CNN model includes five convolutional layers. And when different conventional convolution layers are replaced in fully-convolutional Siamese architecture, the tracker DOSiam has different tracking results.

To conduct a comparison experiment with fully convolutional subnetwork, we train the proposed algorithm with conventional and depthwise over-parameterized convolutional layers. Extensive testing experiments are conducted on five tracking benchmarks. Experimental results demonstrate that DO-Conv is more beneficial to capture a variety of appearance changes of the targets. In the feature extraction subnetwork, named convolutional layers are transformed into the specific layers that combine depthwise with conventional convolution layers. On OTB2015 [51], we evaluate the precision and success of DOSiam, where the DO-Conv is placed in different convolutional layers. The result of ablation study are shown in Table 1. The tracker DOSiam with depthwise over-parameterized convolutional layer improves tracking precision and success rate. When the DO-Conv is used in the second place, DOSiam achieves the best tracking performance, and the second tracking performance in the third place. In Siamese network framework, the proposed feature extraction subnetwork enhances the expression ability of target appearance and improves targets robustness among similar distractors. The proposed tracking algorithm avoids disturbing influences of the target fast motion, in-plane rotation, motion blur, scale variation and other challenging factors.

**Table 1:** Ablation study on different convolution layers. DOSiam achieves the best tracking performance when the DO-Conv is placed in the second layer.

Layer					OTB2015	
1	2	3	4	5	Prec	Suc
Baseline					0.771	0.582
√					0.695	0.544
	√				0.810	0.597
		√			0.774	0.582
			√		0.768	0.570
				√	0.545	0.414
√	√				0.671	0.523
√		√			0.683	0.536
√			√		0.629	0.488

### 4.3 Evaluation on OTB2015

OTB2015 is a classic benchmarks, which includes 100 sequences. It has eleven interference attributes including background clutters (BC), fast motion (FM), illumination variation (IV), scale variation (SV), out-of-view (OV), occlusion (OCC), motion blur (MB), in-plane rotation (IPR), out-of-plane rotation (OPR), low resolution (LR), and deformation (DEF). The standard evaluation metrics of OTB are success rate and precision. For each frame, we calculate the overlap rate between ground truths and bounding boxes. We evaluate the success rate by different intersection over union (IOU) thresholds. The frame is considered to be tracking successfully if the overlap rate is greater than a threshold. Through evaluation, a success plot can be obtained. The precision plot can be received in a similar way.

**Fig 3:**
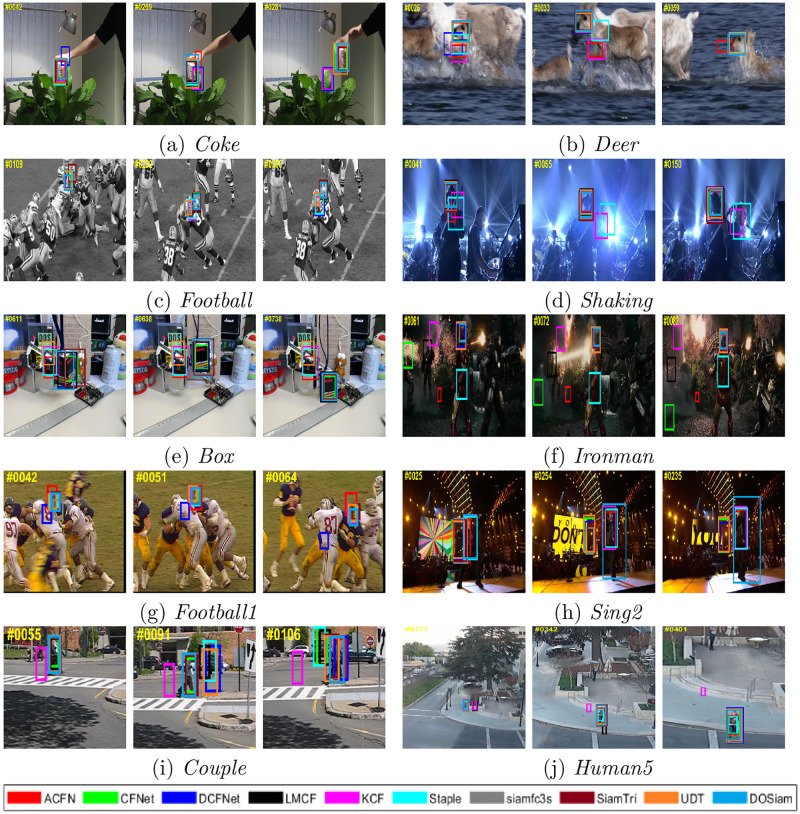
Comparison with state-of-the-art tracking algorithms. The tracker DOSiam gets best tracking results in challenging environments of fast motion, scale variation, motion blur, in-plane and out-plane rotations.

The tracking performance comparison results of other challenging videos are shown in the Fig 3. In the *coke* sequence, our tracker accurately locates target. KCF and DCFNet [52] algorithms undergo the tracking drift when trackers track the target of fast movement. In the *football* sequence, ACFN [53] and DCFNet [52] algorithms might perform poorly when the target undergoes significant occlusion and background clutters. However, our tracker separates background and targets from the background clutters and accurately estimate the target bounding box.

**Fig 4:**
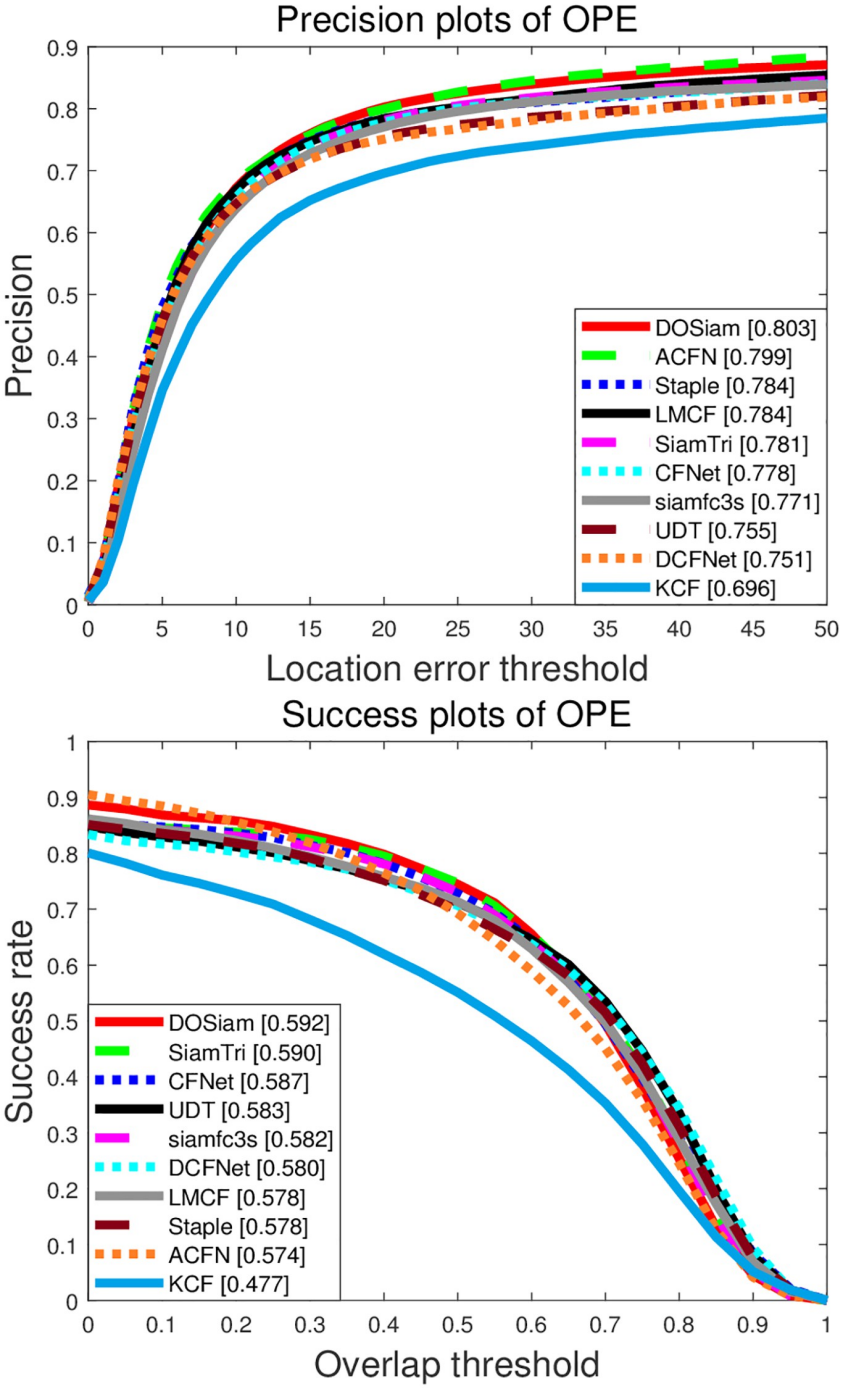
Precision and success plots on OTB2015.

**Fig 5:**
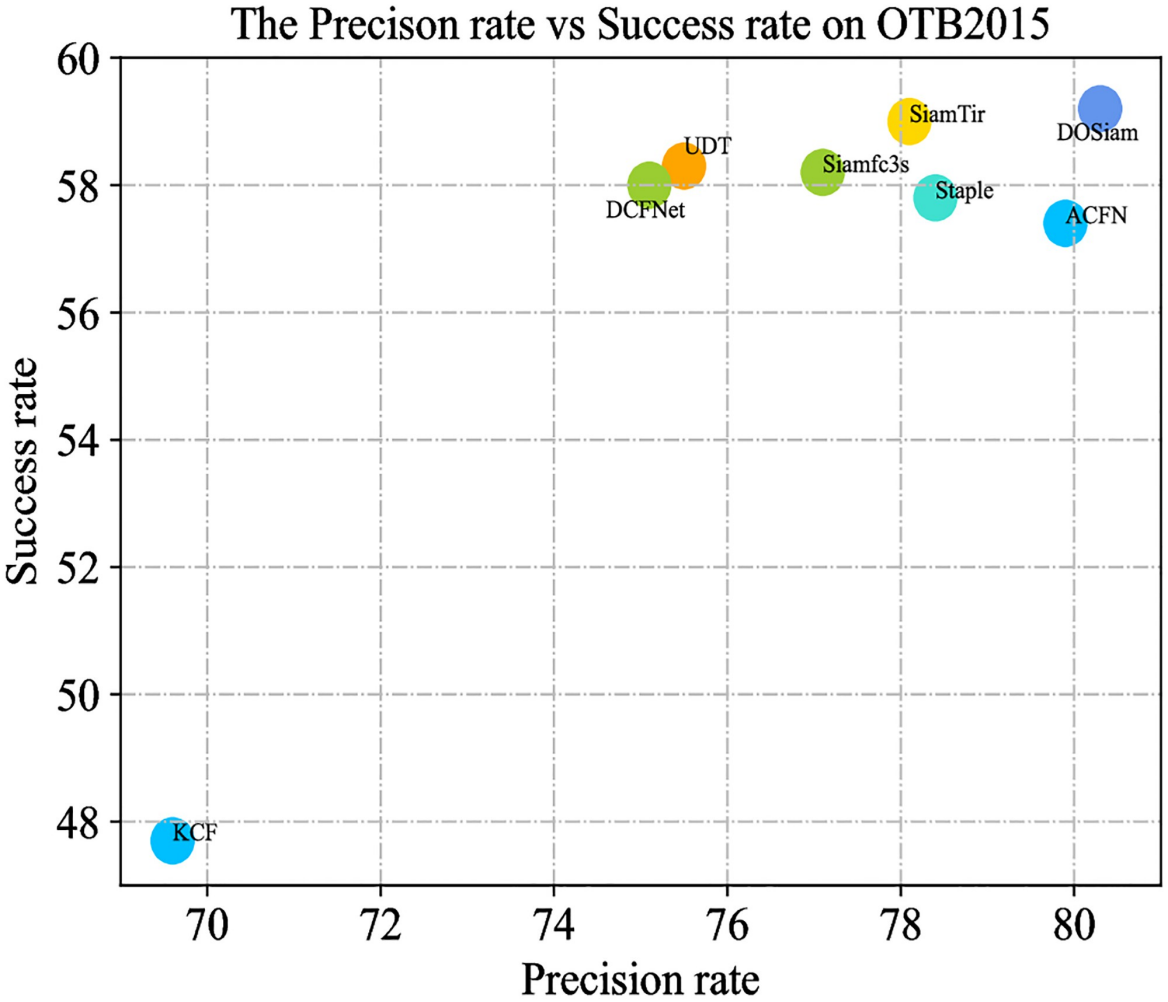
Precision and success rate on OTB2015.

We describe the evaluation results with the success and precision of our algorithm against with the state-of-the-art trackers. In this experiment, we compare our DOSiam with state-of-the-art trackers, including ACFN [53], LMCF [54], Staple [55], SiamFC-tri [56], CFNet [16], siamfc3s [9], DCFNet [52], UDT [57] and KCF [7]. The success and precision plots of these trackers are shown in Fig 4. The tracker DOSiam demonstrates outstanding result in success rate and precision and the result of DOSiam and state-of-the-art trackers are shown in Fig 4. The precision and success of DOSiam are 4.8% and 0.9% higher than UDT. The precision and success of DOSiam are 2.2% and 0.2% higher than SiamTri. By comparing of DCFNet, the precision is improved by 5.2% from 75.1% to 80.3%. Also, in Fig 5, we present the tracking results in terms of the precision and success rates on OTB2015.

**Fig 6:**
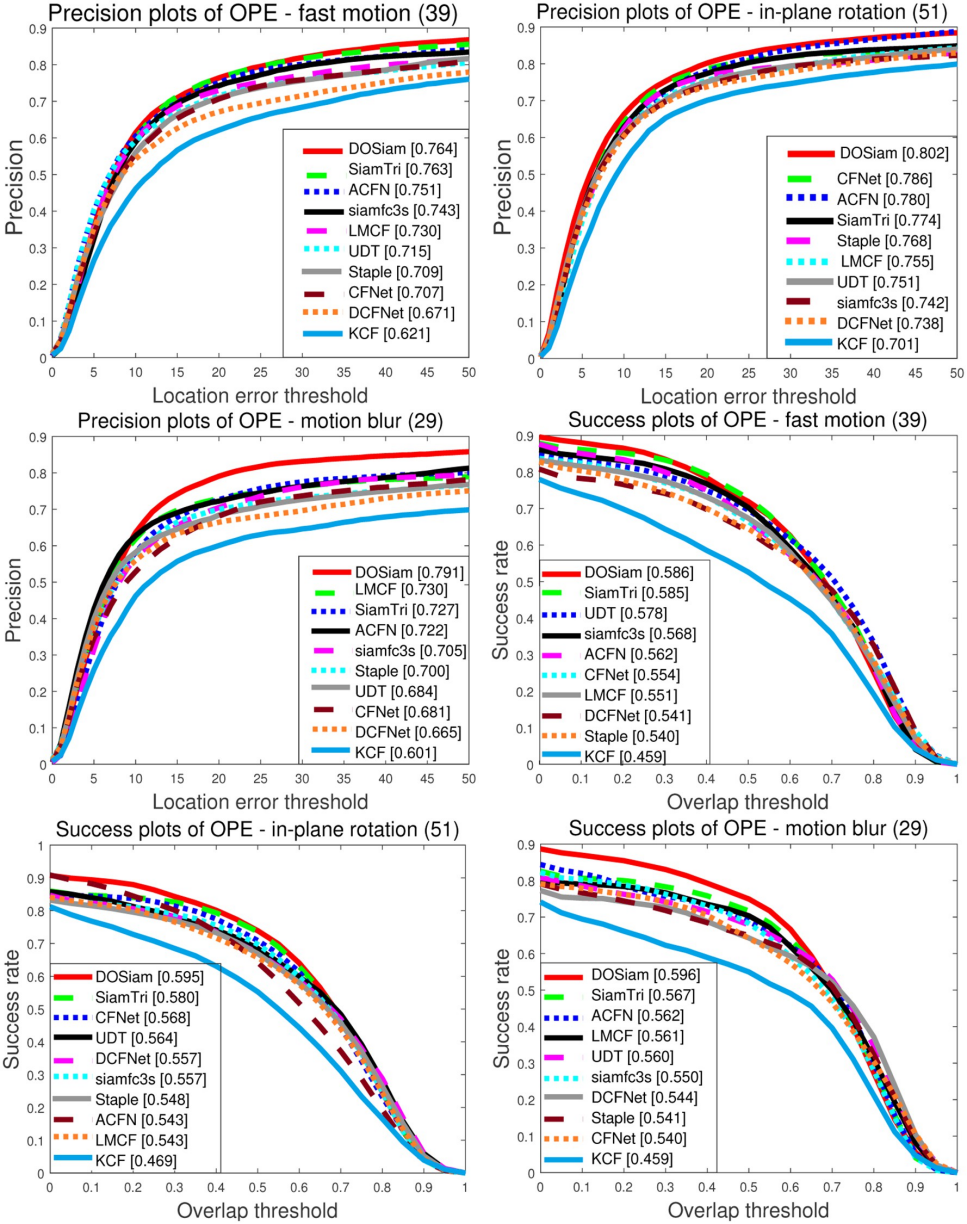
Precision and success plots of OPE. Our tracker has good tracking performance in challenge environments fast motion, in-plane rotation, motion blur and the success rate and precision rate in these challenge environments are OTB2015 benchmark tested.

**Fig 7:**
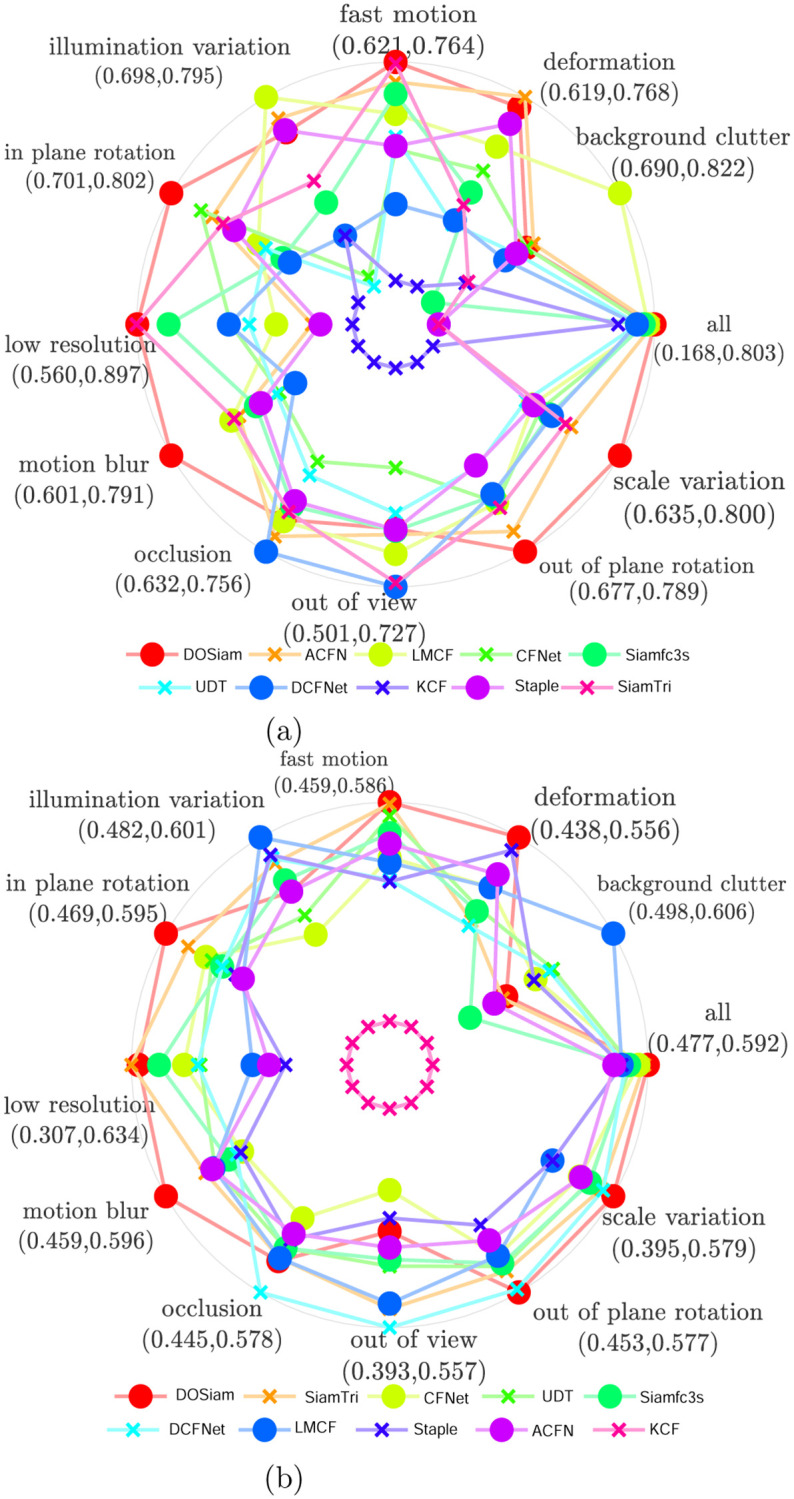
A comparison of the DOSiam with state-of-the-art trackers in terms of success rate and precision on OTB2015 with different attributes.

**Fig 8:**
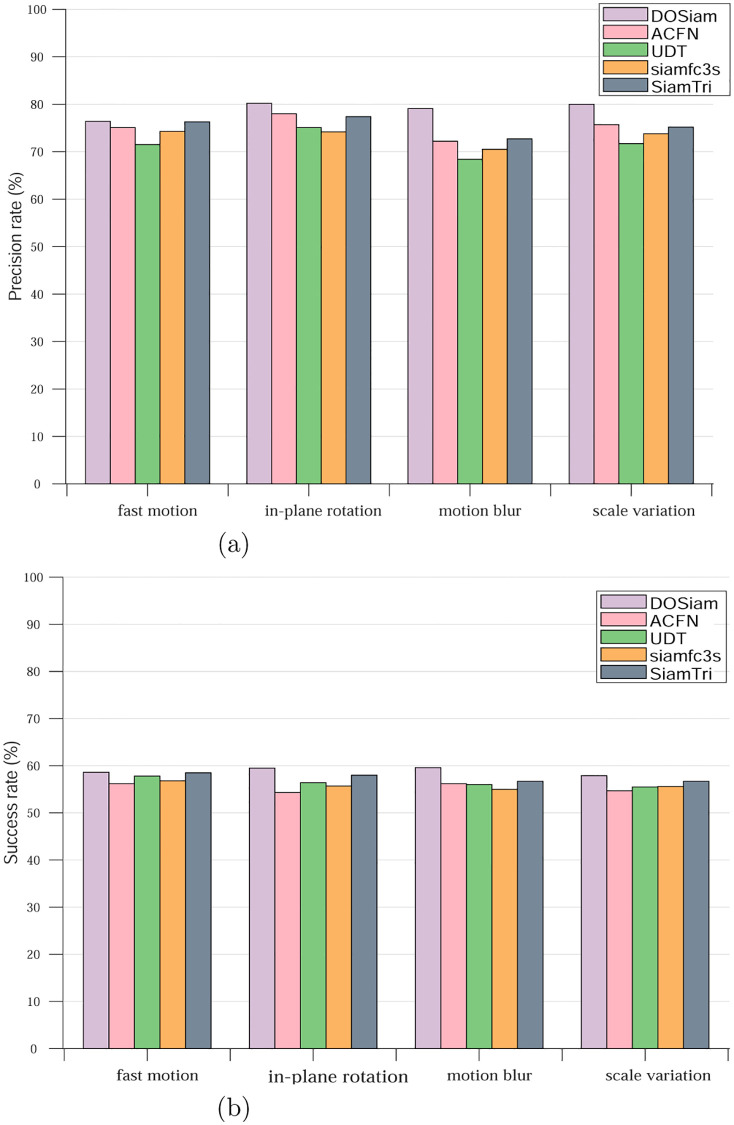
A comparison of the DOSiam with state-of-the-art trackers of precision and success rate in terms of fast motion, in-plane rotation, motion blur and scale variation.

Compared with other tracking algorithms, it can be seen from the Fig 6 that the DOSiam is able to handle various challenging factors, when undergo the challenging factors such as fast motion, motion blur, out of plane rotation and scale variation. To validate robustness of our tracker, we analyse the results of challenging factors in different conditions, such as, illumination variation, scale variation, occlusion, motion blur, fast motion and so on. Fig 7 (a) represents the largest precision and minimum precision values of the tracking algorithm in eleven attributes. We note that the tracker DOSiam is sensitive to motion blur and scale variation on OTB2015. The Fig 7 (b) represents the success value of our algorithm and other tracking algorithms where the maximum and minimum of the success rate for the tracking algorithms are represented. From Fig 8 (a), we can see that our tracker achieves higher precision rate in terms of fast motion, in-plane rotation, motion blur and scale variation. From Fig 8 (b), we can see that our tracker achieves higher success rate in terms of fast motion, in-plane rotation, motion blur and scale variation.

### 4.4 Evaluation on VOT2016

The visual object tracking (VOT) benchmark contains many versions of the datasets. VOT2016 [58] dataset contains 60 testing sequences. In the case of the ground truth and the bounding box has no overlap, the tracker will be initialized after five frames. The tracking performance is evaluated in terms of accuracy (A), robustness (R) and expected average overlap (EAO). Accuracy (A) calculates the average of the intersection ratio of the whole video sequence. Robustness (R) is the number of tracking failure frames and the failure rate can be calculated by robustness. It is used to evaluate the stability of the tracker.

**Fig 9:**
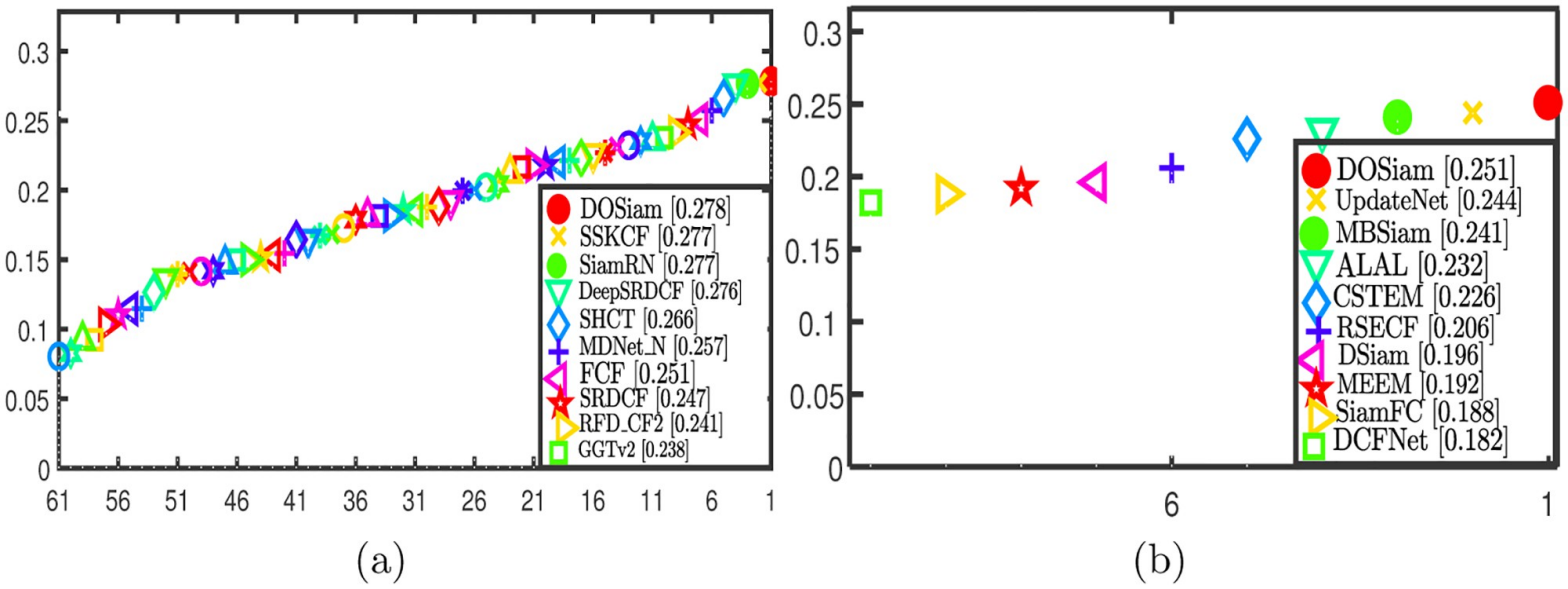
A comparison of the DOSiam with state-of-the-art trackers in terms of success rate and precision on VOT2016 and VOT2018 with different attributes, respectively.

We test our DOSiam tracker and nine state-of-the-art methods on VOT2016. The tracker DOSiam achieves the top-ranked performance on expected average overlap. The comparison algorithms that we use are SiamFC [9], DeepSRDCF [59], SRDCF [5] and etc. The Fig 9 (a) shows the tracking comparison results on VOT2016. In terms of expected average overlap, robustness and accuracy, we compare our tracker algorithm in Table 2. The tracker DOSiam has the top-ranked with EAO on VOT2016 testing datasets. The EAO of DOSiam is 7.5% higher than SiamFC. DOSiam achieves a gain of 0.1% in EAO compared to SiamRN. Among the top two trackers in the expected average overlap with VOT2016, they are based on deep features to locate targets.

**Table 2:** Comparison with state-of-the-art trackers on VOT2016 in terms of EAO, A and R.

Tracker	EAO	A	R
*SiamFC*	0.203	0.511	0.421
*DeepSRDCF*	0.276	0.528	0.326
*SRDCF*	0.247	0.535	0.419
*MDNet_N*	0.257	0.541	0.337
*SiamRN*	0.277	0.549	0.382
*SiamAN*	0.235	0.532	0.461
*SHCT*	0.266	0.547	0.396
*FCF*	0.251	0.554	0.457
*SSKCF*	0.277	0.547	0.373
***DOSiam***	**0.278**	**0.530**	**0.370**

### 4.5 Evaluation on VOT2018

VOT2018 [60] also contains 60 testing sequences. The tracking performance is evaluated in terms of accuracy (A), robustness (R) and expected average overlap (EAO). VOT2018 is evaluated in the same way as VOT2016 with several challenging factors including scale variation and occlusion and etc. Expected average overlap (EAO) is evaluation metrics and is estimated for a selected range of sequence lengths. The higher the accuracy (A) and expected average overlap (EAO) scores, the better the tracking performance.

We compare our tracking algorithm with UpdateNet [61], DSiam [19], DCFNet [52], SiamFC [9], MEEM [62] and etc. The result with EAO of DOSiam and state-of-the-art trackers are shown in Fig 9 (b). Through our experiments, we can get detailed spatial information of targets by depthwise over parameterization of convolutional layer. Table 3 explains the effectiveness of our innovation. Effective feature extraction subnetwork can improve the accuracy of tracking. After testing by VOT2018, we can see the values of EAO, A and R for our tracker and the value of EAO is better than baseline.

**Table 3:** Comparison with state-of-the-art trackers on VOT2018 in terms of EAO, A and R.

Tracker	EAO	A	R
*UpdateNet*	0.244	0.518	0.454
*MBSiam*	0.241	0.529	0.443
*DensSiam*	0.174	0.462	0.688
*DSiam*	0.196	0.512	0.646
*MEEM*	0.192	0.463	0.534
*SiamFC*	0.188	0.503	0.585
*Staple*	0.169	0.530	0.688
*RSECF*	0.206	0.470	0.501
*DCFNet*	0.182	0.470	0.543
***DOSiam***	**0.251**	**0.515**	**0.431**

In Table 3, we compare our tracker algorithm with the state-of-the-art trackers in terms of expected average overlap, robustness and accuracy. The tracker DOSiam has the top-ranked with EAO on VOT2018 testing datasets. DOSiam achieves a gain of 0.7% in EAO compared to UpdateNet. By replacing conventional convolutional layer with DO-Conv, the EAO is improved by 6.3% from 18.8% to 25.1%.

### 4.6 Evaluation on VOT2019-RGBT(TIR)

The VOT2019-RGBT(TIR) [63] dataset includes 60 sequences. All video sequences have been annotated with five attributes, including illumination change, camera motion, motion change, size change and occlusion. We compare DOSiam with SiamFC on VOT2019-RGBT(TIR) in terms of A (Accuracy), R (Robustness), EAO (Expected Average Overlap) and FPS (Frames Per Second). The proposed tracker DOSiam achieves higher accuracy than SiamFC. From Table 4, we can see that DOSiam improves accuracy 1.18% over SiamFC. We also compare DOSiam and SiamFC in terms of accuracy in camera motion, motion change, occlusion and size change. The results are showed in Table 5. From Table 5, we can see that our tracker can distinguish the target and background well and avoid tracking drift.

**Table 4:** Comparison with SiamFC on VOT2019-RGBT(TIR) in terms of EAO, A, R and FPS.

Tracker	EAO	A	R	FPS
*SiamFC*	0.2384	0.5668	0.533	79.3869
*DOSiam*	0.2361	0.5786	0.617	80

**Table 5:** Comparison with SiamFC in terms of accuracy in some attributes on VOT2019-RGBT(TIR).

	camera motion	motion change	occlusion	size change
*SiamFC*	0.5683	0.5749	0.4512	0.5668
***DOSiam***	**0.5586**	**0.5780**	**0.4736**	**0.5736**

### 4.7 Evaluation on GOT-10k

GOT-10k includes the training set and test set, and includes 84 target categories and 32 movement modes. The target classes in training set and test set are zero-overlapped. We compare our tracker with some state-of-the-art trackers on GOT10k. We choose average overlap (AO) and success rate (SR) as our evaluation criteria. The AO denotes the average overlaps of groundtruth and estimated bounding boxes. The SR0.5 measures the percentage of successfully tracked video frames that the overlaps exceed a threshold 0.5.

**Fig 10:**
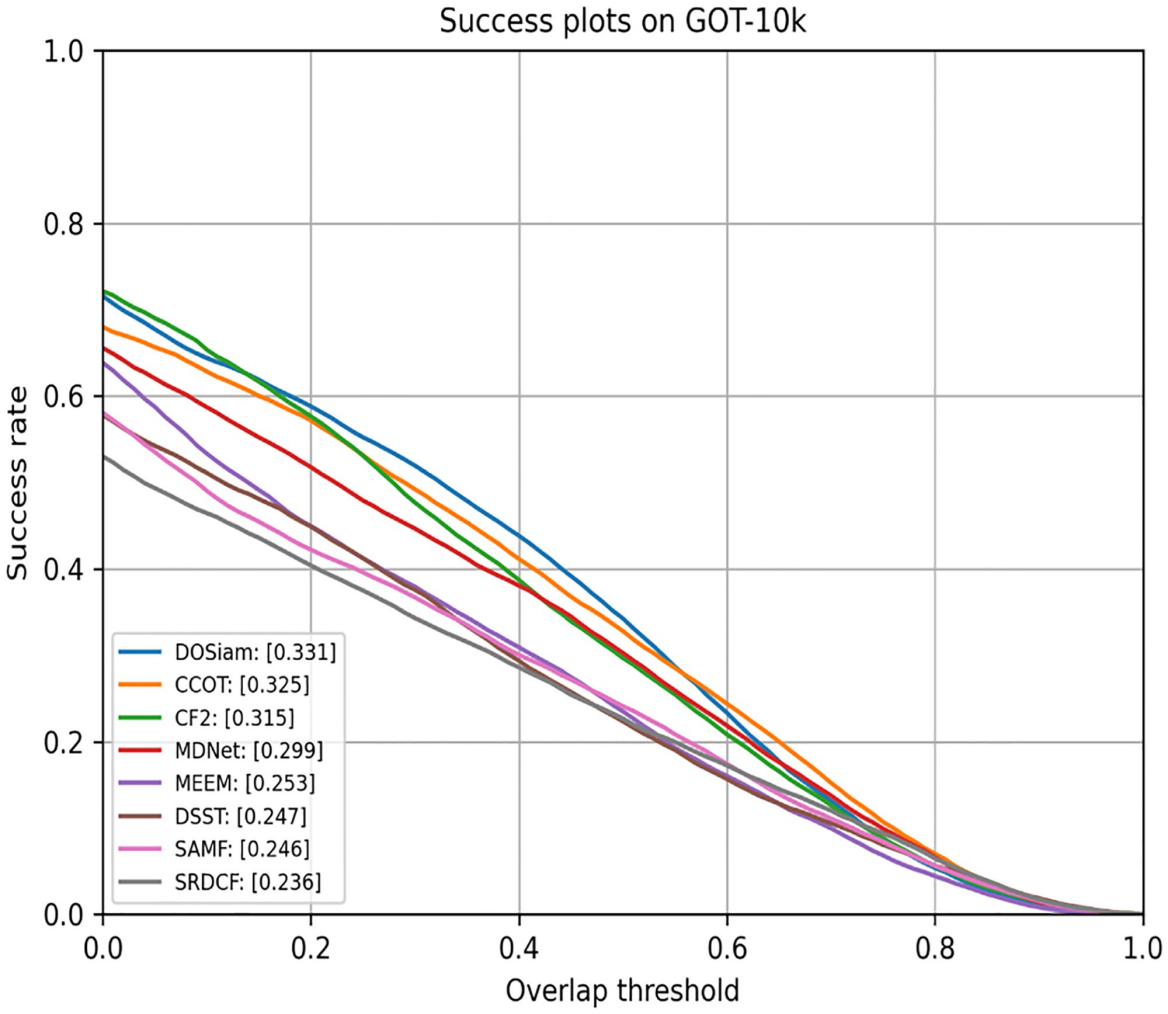
A comparison of the DOSiam with state-of-the-art trackers in GOT-10k.

We submit the results of our tracker DOSiam and some trackers tracking results to the official evaluation server. We show the tracking results in Fig 10 and Table 6. We compare our tracker with some trackers, such as CCOT, MDNet, MEEM, DSST, SAMF, SRDCF and so on. From Fig 10, we can see that our tracker achieves superior tracking performance on GOT-10k test set.

**Table 6:** Comparison with state-of-the-art trackers on GOT-10k in terms of AO and SR0.5.

Tracker	AO	SR0.5
*CCOT* [64]	0.325	0.328
*CF2* [65]	0.315	0.297
*DSST* [8]	0.247	0.223
*MDNet* [15]	0.299	0.303
*MEEM* [62]	0.253	0.235
*SAMF* [66]	0.246	0.241
*SRDCF* [5]	0.236	0.227
***DOSiam***	**0.331**	**0.343**

## 5 Conclusion

In this paper, we propose a novel and high-performance Siamese network tracking framework based on a Depthwise Over-parameterized Convolutional Layer. The DOSiam extracts the target features by DO-Conv that includes the depthwise convolution and conventional convolution. DO-Conv replaces conventional convolution layers to make the best of shallow spatial and deep semantic information for expressing targets appearance change. DOSiam boosts the performance of feature extraction on visual tracking. In experiments, DOSiam achieves real-time tracking performance against other state-of-the-art trackers. We evaluate our tracker against the state-of-the-art trackers on five challenging benchmarks. From experimental evaluation, we can see that our tracker achieves a higher tracking success rate than state-of-the-art trackers. Our tracker improves 3.2% in term of precision rate of OTB2015. DOSiam addresses the major challenges and achieves excellent tracking performance.
